# Inter-rater reliability and test-retest reliability of the Performance and Fitness (PERF-FIT) test battery for children: a test for motor skill related fitness

**DOI:** 10.1186/s12887-021-02589-0

**Published:** 2021-03-11

**Authors:** Bouwien C. M. Smits-Engelsman, Eline Smit, Rosemary Xorlanyo Doe-Asinyo, Stella Elikplim Lawerteh, Wendy Aertssen, Gillian Ferguson, Dorothee L. Jelsma

**Affiliations:** 1 Department of Health & Rehabilitation Sciences, Faculty of Health Sciences, University of Cape Town, Cape Town, South Africa; 2 Avansplus, University for professionals, Breda, The Netherlands; 3 Department of Occupational Therapy, College of Health Sciences, University of Ghana, Accra, Ghana; 4 Department of Paediatrics, 37 Military Hospital, Accra, Ghana; 5 Developmental and Clinical Neuropsychology, University of Groningen, Groningen, the Netherlands

**Keywords:** Reliability, Physical fitness, Skill-related physical fitness, Children, Low resourced settings, Psychometric properties, Motor development

## Abstract

**Background:**

The Performance and Fitness (PERF-FIT) test battery for children is a recently developed, valid assessment tool for measuring motor skill-related physical fitness in 5 to 12-year-old children living in low-income settings. The aim of this study was to determine: (1) inter-rater reliability and (2) test-retest reliability of the PERF-FIT in children from 3 different countries (Ghana, South Africa and the Netherlands).

**Method:**

For inter-rater reliability 29 children, (16 boys and 13 girls, 6–10 years) were scored by 2 raters simultaneously. For test–retest reliability 72 children, (33 boys and 39 girls, 5–12 years) performed the test twice, minimally 1 week and maximally 2 weeks apart. Relative and absolute reliability indices were calculated. ANOVA was used to examine differences between the three assessor teams in the three countries.

**Results:**

The PERF-FIT demonstrated excellent inter-rater reliability (ICC, 0.99) and good test-retest reliability (ICC, ≥ 0.80) for 11 of the 12 tasks, with a poor ICC for the Jumping item, due to low spread in values. A significant difference between first and second test occasion was present on half of the items, but the differences were small (Cohen’s *d* 0.01–0.17), except for *Stepping, Side jump and Bouncing and Catching (*Cohen’s *d* 0.34, 0.41 and 0.33, respectively)*.* Overall, measurement error, Limits of Agreement and Coefficient of Variation had acceptable levels to support clinical use. No systematic dissimilarities in error were found between first and second measurement between the three countries but for one item (Overhead throw).

**Conclusions:**

The PERF-FIT can reliably measure motor skill related fitness in 5 to 12-year-old children in different settings and help clinicians monitor levels of fundamental motor skills (throwing, bouncing, catching, jumping, hopping and balance), power and agility.

## Background

Despite the global interest in promoting physical activity and fitness among school-aged children, there is a paucity of studies concerning this topic from developing countries. The few studies available provide data that children living in socioeconomically disadvantaged circumstances are disproportionally experiencing limited opportunities to develop adequate levels of physical fitness [1]. The levels of physical fitness and motor skills in children are important factors to be able to participate in daily activities such as outdoor play and sports. Child factors can partly determine the level of physical fitness or motor skills, but the environment can be as influential [2]. It is reported that South African children living in low-income areas have overall lower levels of aerobic fitness and motor skills compared to children in other settings [3, 4]. For aerobic fitness there seems to be consensus to use the Shuttle Run test as field-based test and data are available for many countries with different socioeconomic levels [5]. Despite the fact that simple field-based tests for fitness are available that could be used in low-income countries, there are still large gaps in the literature. By way of example, two recent studies evaluated temporal trends between 1967 and 2017 in muscular fitness (sit-ups) and handgrip strength in children and adolescents aged 9–17 years. For sit-ups data were available for 9.939.289 individuals from 25 high-income, five middle-income and one low-income country (Mozambique) and for handgrip strength for 2.216.320 children and adolescents from 13 high-income, five middle-income, and one low-income country (Mozambique) [6, 7]. Moreover, Tomkinson’s studies on temporal and secular trends in the Shuttle run data, could only include data from high-income and middle-income countries [8, 9].

One major obstacle to conducting research on motor skill development in children in low-income settings is the lack of accurate and reliable norm referenced measures of motor coordination. The majority of the developmental studies evaluating motor skills use the Movement Assessment Battery for Children first or second edition, or the Bruininks–Oseretsky Test of Motor Proficiency first or second edition [10–14]. Although these well-validated and reliable tests are popular in high-income countries, their utilization in low resource areas is relatively low due to their expensive nature. Moreover, the lack of cross-cultural adaptations of the items and the unavailability of norms for low resource populations make their applicability questionable in those areas. Normative scores provided for an instrument may not be applicable to other populations because they may not reflect the typical cultural motor experiences [15–17].

Given that children in low resource areas are largely underrepresented in motor performance research, it was prudent to develop a new standardized norm-referenced test called Performance and Fitness (PERF-FIT) test battery for this target population that integrates muscular fitness and motor coordination, as this deemed more ecologically valid. The PERF-FIT was developed for health and teaching professionals (occupational and physical therapist, school nurses and physical educators). Consistent with the World Health Organization’s (WHO, 2007) International Classification of Functioning, Health and Disability [18], the PERF-FIT is developed as a tool measuring the “activity” component of the WHO framework rather than the “body structure and function”. The rationale for this focus is the desire to detect deviations in the development of motor skills that have a functional impact on the day-to-day activities of children. The benefit of such a tool is early identification of children with deficits in fundamental movement skills and muscular fitness. The PERF-FIT provides an integrated way to evaluate fundamental motor skills and muscular-fitness that has cross-cultural applicability and can be used in diverse resource-limited environments. Not mastering motor skills and low anaerobic fitness limits children’s ability to participate in everyday activities and sports, but also diminishes children’s motivation to participate in physical activities [19]. The detection of variations in motor competence and anaerobic fitness (together called motor skill related physical fitness: see Table 1) [20] will enable researchers and clinicians to explore possible etiological mechanisms and policy makers to develop preventive measures.

**Table 1:** Items of the PERF-FIT

PERF-FIT	
*Motor Skill Performance items*	
Bouncing and Catching	Children bounce tennis ball to the floor and catch. This series involves five bouncing and catching items of increasing skill difficulty. All children start at the easiest level. This series is discontinued if the child scores less than 6 out of 10 catches.
Throwing and Catching	Children throw tennis ball in the air to at least eye level height and catch. This series involves five throwing and catching items of increasing skill difficulty. All children start at the easiest level of this series. The series is discontinued if the child scores less than 6 out of 10 catches.
Jump	Children are asked to jump inside an agility ladder. This series involves four jumping items of increasing difficulty. Two test trials are allowed if maximum score is not obtained.
Hop	Children are asked to hop inside an agility ladder. This series involves four hopping items of increasing difficulty for each leg. Two test trials are allowed if maximum score is not obtained.
Balance	Children are asked to perform two (2) static balance tasks for each leg and three (3) dynamic balance tasks. Tasks involve knee hugging, grasping the foot and picking cans from the floor at close and far distance.
*Agility and Power items*	
Running	Children are asked to run (one foot per square) in 3.5 m agility ladder and run around a bottle placed at a distance of 50 cm from the starting line and return the same way as fast as possible. Two test trials are given for each child. The time taken (in seconds) to complete this task and number of mistakes made are recorded.
Stepping	Children are made to step with two feet in each square of a 3.5 m agility ladder and run around a bottle placed at a distance of 50 cm from the starting line and return the same place as fast as possible. Two test trials are given for each child. The time taken (in seconds) to complete this task and number of mistakes made are recorded.
Side Jump	Children are required to jump sideways on their feet. One foot per square, in the same three squares of the agility ladder. The total number of correct landings in 15 s is recorded for each of the two test trials.
Long Jump	Children are asked to jump forward as far as possible and land on their feet in a controlled manner (i.e. balanced landing). The distance between the starting line and the heel of the foot that landed closest to the starting line is measured in centimeters. Two test trials are given.
Overhead Throw	Children kneel just behind a starting line and throw a sandbag (2 kg) forward as far as possible. The bag is held over the head and thrown from a starting position behind the head. The distance between the starting line and the part of the sandbag closest to the starting line is measured in centimeters. Two test trials are performed.

Physical fitness is a multi-dimensional concept comprising of a set of attributes that people exhibit, which relates to their level of ability to perform physical activity [21]. Physical fitness is usually divided into two broad categories; health-related and motor skill-related components [20]. Health-related physical fitness refers to those aspects of fitness that have close relationship with positive health outcomes and include body composition, cardiovascular endurance, flexibility, muscular strength and endurance. Motor skill-related physical fitness is made up of aspects of physical fitness that facilitate performance in sports and motor skills [20] and is the construct intended to be measured by the PERF-FIT (see Table 1). Hence, the PERF-FIT not only assesses various fundamental movement skills (locomotor, stability, and object control) but also combines motor coordination and power aspects, which are embedded in the different test items (for instance hopping 4 times sequentially over foams with a height of 5 or 10 cm or throwing a 2 kg sandbag). For a tool that intends to measure motor skill-related physical fitness, integrating these aspects may be more appropriate than testing motor coordination and power aspects in isolation. After having established good content and structural validity of the PERF-FIT [22, 23], the present study examined how consistent scores on the PERF-FIT are under different circumstances and different populations. The purpose of the first part of the study was to check if the item instructions for scoring, would lead to comparable results between two raters, when assessing the performance of the child at the same time (inter-rater reliability).

Next, we evaluated test-retest reliability. Usually, clinical measurement is rarely perfectly reliable as raters and subjects are known to respond with some inconsistency. Since reliability is generally population specific, a comparison of reliability between different populations is advised [24]. Due to expected use of the PERF-FIT in very different contexts, we collected data in three countries with different groups of raters. Subjects of three different populations were tested twice, in order to test the stability of the measure over time.

## Method

### Participants

**Fig. 1:**
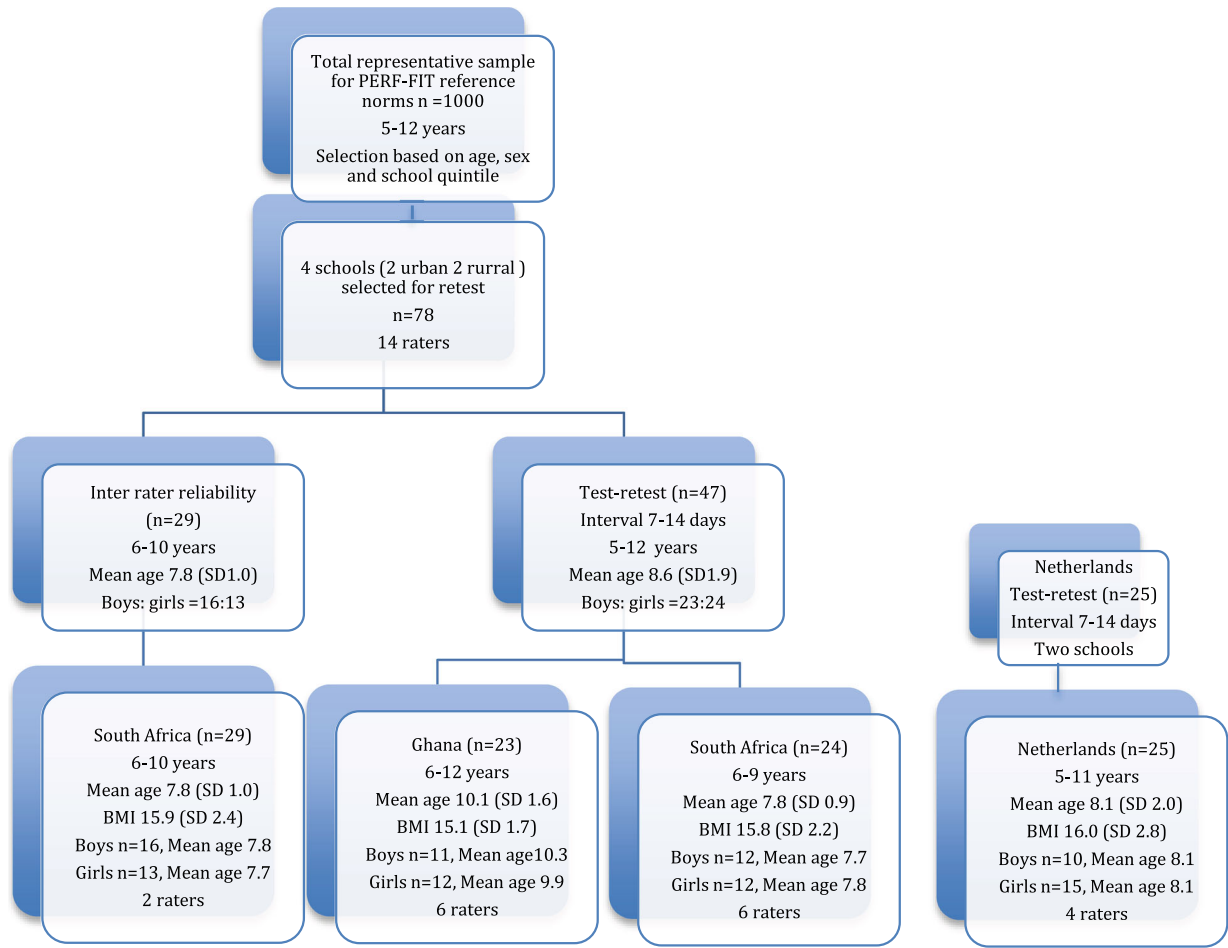
Flow chart for the total study. Countries in which the study took place and the number of participants. Demographic participant information: Number of children per study, Age range, Mean age and Standard Deviation (SD), Mean (SD), Body mass Index (BMI), Ratio Boys and Girls, and number of raters per study

The data for the test-retest study were sampled from a random group of elementary school children included in the data collection study for reference norms that took place in South Africa and Ghana. In that project, population-based sampling based on census data from 2017 was used to recruit 1000 children between 5 and 12 years of age from low SES background. The governmental categorization of schools and its concomitant funding was used for the selection of schools, which is based on the socioeconomic status of the community in which the schools are located. In the current study, sampling was done at two levels: schools and participants. One school was selected in the greater city area (Cape Town and Accra) and one in more rural areas to ensure maximum coverage. Children were randomly picked from a class list by a teacher not involved in the study in each school. Similarly, participants were recruited from two elementary schools in middle-income areas (based on postal codes) in Tilburg, the Netherlands (NL). In total, 101 children between 5 and 12 years of age were recruited (See Fig. 1).

First, we examined the inter-rater reliability of the PERF-FIT test battery. Twenty-nine 6–10-year-old South African children were included in this part of the study. Next, we examined test-retest reliability to evaluate possible variance in performance between two test moments in the children and if the variance was stable under the different testing circumstances. In total, 72 children between 5 and 12 years of age completed the test-retest part of the study; South African children (24), Ghanaian children (23) and Dutch children (25). The ethical review committees of the University of Cape Town, Ghana Health Service and University of Groningen gave their approval for the study (UCT HREC Ref 598/2019: HREC139/2019; GHS-ERC 084/04/19; PSY-1920-S-0107). Demographic characteristics are summarized in Fig. 1.

### Procedure

Permission to approach the head teachers were obtained from the school districts. Verbal and/or written explanations of study purpose, test procedures, benefits and risks were provided to parents. Children were included after parents or caretakers signed the consent forms and children gave assent to participate. Children included were a random sample of school children aged 5–12 years and with understanding of the local language. Children with health-related conditions were excluded based on the Physical Activity Readiness Questionnaire (PAR-Q) [25]. In addition to PERF-FIT scores, data sought included age, height, weight and gender. No other information was available to the raters about the children.

Assessments were performed under the circumstances that the test was developed to be used in; on the school’s premises outside (GH and SA), in the gym or hall (NL and SA) and a physiotherapy practice (NL). Participants completed standardized warm-up procedures prior to testing as prescribed in the manual. They were allowed practice trials for each test item before the scored trial as indicated in the manual. Children, who did not have proper shoes, performed the test barefoot on both occasions. All of the Ghanaian children were tested barefoot; part of the South African children wore uniform shoes and part was barefoot. All the Dutch children wore sneakers.

The lead author trained at least one rater per country but was not present at any of the test sessions. The trained raters instructed the other raters in SA, GH and NL during a half-day training, where they practiced in small groups to obtain a solid routine. Raters, all with at least 3 years working experience with children, were selected as being representative of the future users; pediatric physiotherapists, physiotherapists and occupational therapist, teaching assistants and a school nurse. The raters conducted all the testing during school hours except in the Netherlands where part of the testing was done on a day-off.

### Inter-rater reliability study

One rater administered the test (instruction and scoring), while the other one observed from a distance and scored independently without any communication.

### Test-retest reliability study

To examine the degree to which test scores remained stable when measured on two occasions, we planned three sequential research projects in three different countries. In SA and GH, data were sampled for validation purposes, of which a group randomly selected children was tested twice. The Dutch sample was added to evaluate if testers and children from a different context would influence the test-retest reliability. The re-test assessments followed between seven and fourteen days after the first test.

### Outcome measure: PERF-FIT

The PERF-FIT measures motor skill related physical fitness in children aged between 5 and 12 years old. The test has two subscales: a Performance part and a Fitness part. See Table 1. The PERF-FIT test battery is easy to administer, low-cost and developed for measuring performance-related physical fitness in school-aged children living in low-income settings and has excellent content validity and good structural validity (Smits-Engelsman et al., 2020a, 2020b). A full description of the PERF-FIT test battery is available through the first author [26].

### Agility and power *subscale*

This subscale contains five items: *Running, Stepping, Side Jump, Long Jump, and Overhead Throw*. For the *Agility and power subscale* children perform two trials for each item and get 15 s rest in between.

### Motor skill performance *subscale*

This subscale contains five Skill Item Series (SIS) of increasing difficulty; *Bouncing and Catching, Throwing and Catching, Jumping, Hopping* (left and right), and *Balance*. All children start at the easiest level and a series is terminated when they do not reach the criterion number of points for the item after two trials. If a child obtains the maximum number of points after the first trial no second trial is given and the child proceeds to the next level of difficulty.

After the first round of collecting validity data in Brazil [22], it was found that most children obtained a maximum score on the static balance series and it was decided to increase the total number of seconds of the static balance series from 40 to 60 s for future studies. At this moment the data collection for SA had already started with the 40 s protocol. Therefore, the South African data on one item, *Static balance,* was discarded in the current paper. This was the only adaptation in the protocol, which then was used for data collection in GH and NL.

### Data analysis

For the evaluation of measurement properties of the PERF-FIT, we followed the international consensus on terminology and definitions of measurement properties as suggested in the COnsensus- based Standards for the selection of health Measurement Instruments (COSMIN) [27]. Relative reliability, which is the degree to which individuals maintain their position in a sample over repeated scoring or testing, was determined by calculating the two-way random intra-class correlation coefficient (ICC 2,1a) for absolute agreement of single measures. The 95% confidence interval (CI) was calculated for each ICC [28]. Reliability was considered poor for ICC values < 0.40, fair for values between 0.40–0.59, good for values between 0.60–0.74, and excellent for values between 0.75–1.00 [29, 30]. ICC values above 0.75 were considered acceptable for test-retest reliability [31].

Bias corrected and accelerated (BCa) bootstrapping interval was used for the paired samples t-tests to compare the means of test (T1) and retest (T2) in order to evaluate whether there was any statistically significant bias between the test results and to calculate confidence intervals based on 5000 samples. Bootstrapped confidence intervals are more robust as they are less affected by the distribution of the scores [32].

Cohen’s *d* effect sizes (d) were calculated to determine the practical significance of these differences. Values greater than 0.5 were taken to indicate a moderate effect and values greater than 0.8 were taken to indicate a large practical significance [33].

Next, indicators of absolute reliability were calculated to determine the degree to which repeated measurements vary for individuals, expressed in the actual units of measurement, or as a proportion of the measured values. The Standard Error of Measurement (SEM), Bland and Altman’s 95% Limits of Agreement (LoA) [34] and coefficient of variation (CV) are all measures of absolute reliability that were used in this study.

The calculation of SEM and LoA do not depend on sample size, but the precision of their estimate for the population parameter does. Bland and Altman recommended sample sizes of at least 50 individuals in a study to consider the sample LoA to be a precise estimate of the population LoA [34]. Since we were also interested in a group comparison we oversampled, and aimed at 25 subjects per country.

The SEM, as measure of precision of the assessment, was determined using the ICC through the formula SEM_agreement_ = SD*√(1-ICC_agreement_) in which SD is the sample SD of the grand mean and ICC is the calculated intraclass correlation coefficient [35].

Minimal Detectable Change (MDC) was calculated as MDC_95_ = 1.96* √2 * SEM _agreement_ [36]. The MDC_95_ is the minimal amount of change observed before the change can be considered to exceed the variation and measurement error at the 95% confidence level.

Absolute reliability statistics were also calculated using the standard deviation of test-retest differences (SD_differences)_ and its derivatives. SD_differences_ is the SD of the differences between values on T1 and T2.

The 95% LoA were calculated as the mean difference ± (1.96*SD_differences_) [34, 36].

The Coefficient of Variation (CV) or relative standard deviation is the individual SD expressed as a percent of the mean of T1 and T2 using the formula (SD/Mean) *100. The higher the SD, the greater the percentage of the mean and the higher the %CV. A %CV of < 10% is considered excellent, 10–20% medium, implying good precision, 20–30% high, meaning low precision and > 30% is considered very high, indicating very low precision [37].

To test for possible dissimilarities in the degree of the error between T1 and T2 in the participating countries an ANOVA was run on the difference score (T1-T2) for all items with country (3) as between group factor and post hoc Bonferroni tests.

Statistical data analyses were carried out using SPSS version 25.0. A value of *p* < .05 was considered statistically significant in all analyses.

## Results

### Inter-rater reliability

Very high ICC’s were found ≥0.98 for all items. The results of the inter-rater reliability (*n* = 29) of the two raters are shown in Table 2.

**Table 2:** Inter-rater reliability of the PERF-FIT with Interclass Correlation Coefficient (ICC) and 95% confidence interval (CI) per item

PERF-FIT Items	Inter-rater reliability		
	ICC	95% CI Low	95% CI High
Item 1 Running (s)	0.995	0.990	0.998
Item 2 Stepping (s)	0.980	0.945	0.991
Item 3 Side jump (#)	0.997	0.995	0.999
Item 4 Long jump (cm)	1.00	1.00	1.00
Item 5 Overhead throw (cm)	0.997	0.993	0.999
Item 6 Bounce and Catch (#)	0.999	0.998	0.999
Item 7 Throw and Catch (#)	1.00	1.00	1.00
Item 8 Jump (#)	0.993	0.992	0.998
Item 9a Hop Left (#)	0.993	0.986	0.997
Item 9b Hop Right (#)	0.997	0.995	0.999
Item 10a Static balance (s)	0.986	0.971	0.994
Item 10b Dynamic balance (#)	0.994	0.988	0.997

s: items measured in seconds; cm: measured in centimeters; #: measured in number of times.

### Test-retest reliability

Test-retest reliability results (*n* = 72) of the sixteen raters in the three countries are depicted in Tables 3 and 4. Comparison between first and second test occasion showed that there was a statistically significant difference on half of the items (for t-test results see Table 3). However, this systematic bias was small (Cohen’s *d* 0.01–0.17), except for *Stepping, Side jump and Bouncing and Catching (*Cohen’s *d* 0.34, 0.41 and 0.33, respectively)*.*

**Table 3:** Test-retest reliability with means and 95% confidence interval of PERF-FIT variables at Time 1 and 2. Results of the paired samples t-test with bootstrapping (Mean difference between time 1 and 2 with bias, standard error, corrected 95% confidence intervals of the mean difference, t- and *p*-values and Cohen’s d effect size), results of the ANOVA (F and *p-*values of the comparison of the reliability results between the three countries)

PERF-FIT items	Time 1 Mean	95% CI Lower	95% CI Upper	Time 2 Mean	95% CI Lower	95% CI Upper	Mean Difference	Bias	Std. Error	BCa 95% CI Lower	BCa 95% CI Upper	t-value	*p-*value	Effect size	F-value	*p-*value
Item 1 Running (s)	7.14	6.86	7.43	7.22	6.91	7.53	−0.08	0.00	0.12	−0.32	0.15	−0.69	0.50	−0.06	0.84	0.44
Item 2 Stepping (s)	14.19	13.54	14.84	13.29	12.70	13.87	0.90	0.00	0.24	0.44	1.37	3.77	0.00	0.34	2.50	0.09
Item 3 Side jump (#)	25.26	23.50	26.93	28.57	26.58	30.51	−3.31	0.00	0.44	−4.18	−2.46	−7.50	0.00	−0.41	0.01	0.99
Item 4 Long jump (cm)	120.14	114.03	125.87	120.57	115.60	125.53	−0.43	− 0.04	1.73	−3.78	2.90	−0.25	0.81	−0.02	0.70	0.50
Item 5 Overhand (cm)	212.19	198.86	226.06	212.90	214.29	199.50	−0.71	−0.02	3.17	−7.26	5.46	−0.22	0.83	−0.01	4.48	0.02
Item 6 Throw and catch (max 50#)	36.40	32.99	39.51	38.22	35.06	41.14	−1.82	−0.02	0.62	−3.04	−0.67	−2.93	0.00	−0.13	1.21	0.30
Item 7 Bounce and catch (max 50#)	39.03	35.75	41.83	43.11	40.39	45.31	−4.08	0.00	0.67	−5.52	−2.78	−6.08	0.00	−0.33	1.58	0.21
Item 8 Jump (max 20#)	19.08	18.64	19.44	19.61	19.35	19.82	−0.53	0.00	0.19	−0.93	−0.15	−2.74	0.01	−0.11	2.21	0.12
Item 9a Hop Right (max 20#)	14.96	13.40	16.31	16.01	14.75	17.21	−1.06	0.00	0.36	−1.78	−0.35	−2.92	0.01	−0.11	0.47	0.63
Item 9b Hop Left (max 20#)	13.89	12.17	15.43	14.61	13.06	16.10	−0.72	0.00	0.46	−1.65	0.15	−1.56	0.13	−0.17	0.10	0.91
Item 10a Static balance (max 60s) $	54.85	51.38	57.80	55.83	52.42	58.67	−0.98	0.01	1.02	−2.06	0.93	−0.96	0.35	−0.09	0.40	0.53
Item 10b Dynamic balance (max 32#)	26.18	24.54	27.54	26.63	25.32	27.82	−0.44	0.01	0.45	−1.35	0.46	−0.98	0.35	−0.04	2.59	0.08

Bootstrap results are based on 5000 bootstrap samples; *CI* Confidence Interval, *BCa* Bias Corrected and accelerated bootstrap; $: Data from Ghana and the Netherlands; s: item measured in seconds; cm: item measured in centimeters; #: item measured in number

**Table 4:** Test-retest reliability outcomes of the PERF-FIT item scores. Grand mean of Time 1 and 2, Intraclass Correlation Coefficient (ICC) with 95% Confidence Interval, Standard Error of Measurement (SEM), Minimal Detectable Change (MDC_95_), Mean difference (Mean Dif) between test occasion (SD), Limits of Agreement (LoA) with upper and lower limit, percentage Coefficient of Variation (CV)

PERF-FIT items	Grand Mean	ICC Test-retest	ICC 95% CI	SEM	MDC_95_	Mean Dif 1–2	SD Dif	Lower limit	LoA	Upper limit	CV
Item 1 Running (s)	7.18	0.82	0.71–0.89	0.50	1.39	−0.08	1.01	−2.06	1.98	1.90	7.3
Item 2 Stepping (s)	13.74	0.80	0.64–0.89	1.09	3.01	0.90	2.01	−3.04	3.94	4.85	8.5
Item 3 Side jump (#)	26.92	0.90	0.57–0.96	2.5	6.81	−3.3	3.81	−10.77	7.46	4.16	9.5
Item 4 Long jump (cm)	120.35	0.90	0.84–0.94	7.3	20.36	−0.4	14.69	−29.22	28.79	28.36	6.3
Item 5 Overhead throw (cm)	212.55	0.95	0.92–0.97	13.0	36.17	−0.7	27.06	−53.75	53.04	52.33	6.9
Item 6 Throw and catch (max 50#)	37.31	0.96	0.92–0.97	2.7	7.37	−1.8	5.41	−12.42	10.60	8.78	13.1
Item 7 Bounce and catch (max 50#)	41.07	0.92	0.73–0.96	3.4	9.33	−4.1	5.79	−15.43	11.34	7.26	11.2
Item 8 Jump (max 20#)*	19.35	0.36	0.01–0.59	0.8	2.35	−0.5	1.67	−3.80	3.27	2.74	3.5
Item 9a Hop Right (max 20#)	15.49	0.92	0.86–0.95	1.6	4.37	−1.1	3.12	−7.16	6.11	5.05	13.6
Item 9b Hop Left (max 20#)	14.25	0.90	0.84–0.94	2.0	5.62	−0.7	3.99	−8.54	7.81	7.09	21.0
Item 10a Static balance (max 60s)$	55.34	0.88	0.79–0.93	3.5	9.70	−1.0	7.10	−14.89	13.92	12.94	5.3
Item 10b Dynamic balance (max 32#)	26.40	0.88	0.81–0.92	1.9	5.34	−0.4	3.88	−8.05	7.61	7.16	8.6

*% Agreement for score +/− 1 point =84.7%. $ Data from Ghana and the Netherlands. Max: maximum score. s: item measured in seconds; cm: item measured in centimeters; #: measured in number

Overall test-retest reliability was good to excellent on 11 of the 12 items; all ICC’s were .80 or higher (Table 4). Only the item *Jumping* showed a low ICC due to lack of spread in the data. This was the easiest item and many children had a maximum score (63 and 78% in T1 and T2, respectively). Percentage agreement plus or minus 1 point was 84.7% and the effect size of the difference was small (Cohen’s *d* 0.11). The SD of the differences in scores between the two test occasions and LoA for each variable with its 95% confidence interval are also shown in Table 4. The mean %CV is 9.6%, which indicates excellent stability and the highest %CV (21% for *Hopping* on left foot) was still considered acceptable.

### Comparison per country

**Fig. 2:**
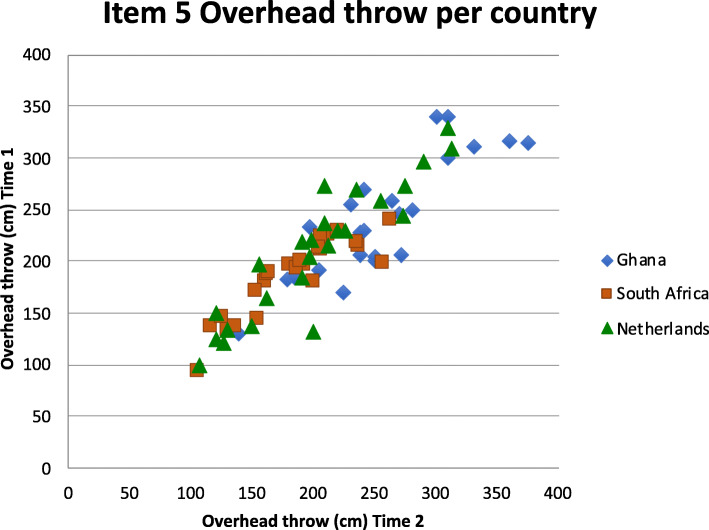
Scatterplot of the mean values (cm) obtained by the children for the Overhead throw at Time 1 (test) and Time 2 (retest) in the three countries

The repeated measure ANOVA showed that the differences between T1 and T2 were not significantly different between countries for 11 of the 12 scores (for ANOVA results see Table 3). Only the *Overhead throw* difference was larger in the Ghanaian children. Post hoc test showed that they were different from the Dutch children, who were slightly worse on the second test while the Ghanaian children in general performed better the second time on this item (see Fig. 2).

## Discussion

A new tool, the PERF-FIT was developed because none of the currently available motor performance tests for children of elementary school age have reference norms for children in low-income areas or combine fundamental skills and muscular skeletal fitness. The PERF-FIT has cross-cultural applicability and can be used in diverse resource-limited environments. This study aimed to evaluate whether the PERF-FIT is a reliable tool and whether the measurement error is acceptable for practical use. Because widely accepted criteria or guidelines for reliability reporting in the health care and medical fields are lacking [27, 38], we chose for a wide variety of outcomes to evaluate the reliability of the PERF-FIT.

Inter-rater reliability depends primarily on good training of the raters, and on good standardization and description of the tasks [26]. Data in this study indicate that this was the case for the PERF-FIT. Test-retest reliability is highly dependent on the situation or on the state and stability of the participants, and is therefore characterized by larger variability, which was confirmed by our results although the agreement between the first and second test occasion was good. A small learning or familiarization effect was seen in 6 of the 12 items. No systematic differences between test-retest differences were found between the testing sites in the three countries in randomly selected children between 5 and 12 years old, except for 1 item. An average CV of 10% - obtained in the current study- means that, assuming the data are normally distributed, 68% of the differences between tests lie within 10% of the mean of the data [37].

### Inter-rater reliability study

The consistency of two different clinicians rating the PERF-FIT was tested. When establishing inter-rater reliability with two observers, one tests if the instructions for scoring were unambiguous and if this led to similar results. Overall results are excellent (mean ICC 0.99), indicating that the two raters did get the same results for the same subjects. Since the children were selected randomly by the teachers, the results can be generalized for the child population within this age range [39].

### Test-retest reliability study

Test-retest reliability concerns the reproducibility of the observed value when the measurement is repeated in a stable population. Studying reliability may seem straightforward, as it is just a matter of repeating the measurement on a reasonable number of individuals. However, interpreting the findings is less simple and a combination of approaches is more likely to give a true picture of reliability [24]. The type of data (continuous) of the PERF-FIT requires standard error of measurement (SEM) [40, 41] and proportions of agreement within specified limits to provide useful information concerning reliability [40]. Given the ICC’s found in this study, one can assume that the PERF-FIT is a reliable tool. ICC’s for 4 items are 80 or higher and 7 items have an ICC of 90 or higher. The relative nature of the ICC is reflected in the fact that the magnitude of an ICC depends on the between-subjects variability and not only on the magnitude of measurement errors [42]. That is, if subjects differ little from each other (homogeneous sample), ICC values can be low even if trial-to-trial variability is small as shown in the *Jumping* item. This item, which is easy in the current population, showed low ICC but good agreement (85%). It will also be of interest to test the reliability of this item when no maximum score is expected, for instance in young children and with neurodevelopmental disorders. In participants with neurodevelopmental delays the between-subjects variability will change as well as the ICC [43].

The need to perform the test twice will cause performance variability, due to changes in motivation and familiarization with the tasks. Detailed analysis of the *Side jump* data, with good ICC (0.90), showed that five children “improved” ten jumps or more, with a maximum of 13. However, this was not due to instruction or circumstances since the five children came from three different countries. Still these differences cannot be attributed to improved anaerobic fitness, or improved motivation since these children showed no improvement on the other items. Hence this finding points more towards a short-term learning effect or getting the clue of the agility required in this task for some children. We therefore added the recommendation in the manual to offer one extra practice opportunity if the assessor sees that a child is still struggling with the understanding the movement of the Side jump.

*Throwing and catching* series also showed small improvements. Some of the African children were less used to this task, which may have increased the learning effect. Consequently, we will emphasize to consistently use the two practice trials *per level of difficulty*, to reduce the learning effect during the scored trials.

#### Test location

The subject population of interest for the PERF-FIT is the group of children in elementary school age living in low socio-economic circumstances. Children with different lifestyles (level of daily physical activity, participation in structured physical education and sports) and testing in different contexts may respond differently to re-testing of some tasks. Therefore, we gathered data in three countries with many raters (*n* = 16), to analyze the reliability across these different populations and environments making the results clinically more widely applicable [24]. Although the testing was done in a standardized way, raters, sites and children were very different. Still, no country-related bias was found except for the *Overhead throw*, where the difference in scores between the two test occasions was larger in the Ghanaian children. Because scoring this item requires the tester to focus on the landing spot, we advise assessors to have the children throw the bag on sand, dirt floor or grass so the sandbag leaves a landing imprint and on a non-sliding surface when testing indoors. The practice trial given in this task is done with submaximal force to avoid fatigue, which may have decreased the familiarization in the first testing.

Despite the noise and distraction, inherent to testing at the school premises in open space, the test results were considerably stable, which implies that the children were able to attend to the instructions under these circumstances. These findings point to the fact that this test is enjoyable and engaging for the children, which keeps them motivated. For optimal test results distraction should be limited to the minimum and we stress the importance of familiarization with the items. An online PERF-FIT training module will be available and will be helpful in this respect for future assessors. It is to be expected that if children are tested in a more clinical one-on-one situation, the variability between test and retest will be even less.

In this study we choose for a wide variety of outcomes because they all have advantages and disadvantages. Both the SEM and LoA were calculated because they differ in the type of measurement error that they describe and in the coverage probability of the reference interval (0.68 versus 0.95%). If the variability in test-retest outcomes depends on the magnitude of the mean values, the use of a ratio statistic is useful to the researchers. The advantage of CV being unitless is that it can be used to compare different instruments, but this makes it harder to translate results into clinical practice.

### Limitations and future research

Given the way the inter-rater reliability was examined, variability as a result of instruction was not tested. During field-based testing, not all sources of variability can be controlled, therefore the design chosen for this study is close to the context this test was developed for. Results of reliability studies are intended to provide information about the amount of error inherent to a measurement tool in a specific population and context. High ICC’s reflect adequate relative reliability for use of the PERF-FIT in the population that has been investigated. However, measures of reliability are generated by distribution-based methods and are dependent on the mean and variance in the group. The Minimal Detectable Change is very susceptible to increased variance given its formula. Reliability studies should be repeated in the population the instrument will be applied in, since variability may be different in groups on children with known poor motor performance, low levels of fitness, or learning disabilities. Also, the impact of BMI on the scores and the reliability should be investigated in different weight categories. Additionally, studies are needed to evaluate the responsiveness of the PERF-FIT or ability of the test to measure changes after intervention. These remaining psychometric issues will be soon addressed on future papers.

## Conclusion

The present study examined inter-rater reliability and test-retest reliability of the PERF-FIT in a manner that replicates how the test is typically used in the actual everyday context. Inter-rater reliability and test-retest reliability were adequate to support clinical use. Hence, the PERF-FIT was relatively stable over time based on the small differences between the repeated measurements and based on the calculated SEM’s. The Coefficient of Variation on average was 10%, indicating good stability. Hardly any systematic differences were found between the testing sites in the three countries, which supports the use of the PERF-FIT by trained raters from a variety of backgrounds in different contexts.

## Data Availability

The datasets used and analyzed during the current study are available from the corresponding author on reasonable request. The PERF-FIT manual and instruction videos can be accessed free of charge for the intended users after registration via the first author for use in low resource communities.

## Abbreviations

PERF-FIT: Performance and Fitness test battery
ICC: Intraclass Correlation Coefficient
SA: South Africa
GH: Ghana
NL: Netherlands
PAR-Q: Physical Activity Readiness Questionnaire
SEM: Standard Error of Measurement
LoA: Limits of Agreement
CV: Coefficient of Variance
SD: Standard Deviation
SIS: Skills Item Series
MDC: Minimal Detectable Change
ANOVA: Analysis of Variance
DCD: Developmental Coordination Disorder
BMI: Body Mass Index

## References

[1] Valentini NC, Clark JE, Whitall J (2015). Developmental co-ordination disorder in socially disadvantaged Brazilian children. Child Care Health Dev.

[2] Ortega FB, Ruiz JR, Castillo MJ, Sjöström M (2008). Physical fitness in childhood and adolescence: a powerful marker of health. Sweden Int J Obes.

[3] Aertssen W, Bonney E, Ferguson G, Smits-Engelsman B (2018). Subtyping children with developmental coordination disorder based on physical fitness outcomes. Hum Mov Sci.

[4] Pienaar AE (2004). Developmental co-ordination disorder in an ethno-racially diverse African nation: should norms of the MABC be adjusted?. J Hum Mov Stud.

[5] Olds T, Tomkinson G, Léger L, Cazorla G (2006). Worldwide variation in the performance of children and adolescents: an analysis of 109 studies of the 20-m shuttle run test in 37 countries. J Sports Sci.

[6] Kaster T, Dooley FL, Fitzgerald JS, Walch TJ, Annandale M, Ferrar K, Lang JJ, Smith JJ, Tomkinson GR (2020). Temporal trends in the sit-ups performance of 9,939,289 children and adolescents between 1964 and 2017. J Sports Sci.

[7] Dooley FL, Kaster T, Fitzgerald JS, Walch TJ, Annandale M, Ferrar K, Lang JJ, Smith JJ, Tomkinson GR (2020). A systematic analysis of temporal trends in the handgrip strength of 2,216,320 children and adolescents between 1967 and 2017. Sports Med.

[8] Tomkinson GR, Léger LA, Olds TS, Cazorla G (2003). Secular trends in the performance of children and adolescents (1980-2000). Sports Med.

[9] Tomkinson GR, Lang JJ, Tremblay MS (2019). Temporal trends in the cardiorespiratory fitness of children and adolescents representing 19 high-income and upper middle-income countries between 1981 and 2014. Br J Sports Med.

[10] Henderson SE, Sugden DA (1992). The movement assessment battery for children.

[11] Henderson SE, Sugden DA, Barnett AL (2007). Movement Assessment Battery for Children-2: Examiner’s manual (2nd ed.).

[12] Bruininks RH (1978). Bruininks–Oseretsky test of motor proficiency: Examiner’s manual.

[13] Bruininks RH, Bruininks BD (2005). Bruininks–Oseretsky test of motor proficiency: Examiner’s manual (2nd ed.).

[14] Blank R, Smits-Engelsman BCM, Polatajko HJ, Wilson PH (2012). European academy for childhood disability (EACD): recommendations on the definition, diagnosis and intervention of developmental coordination disorder (long version)*. Dev Med Child Neurol.

[15] Van Waelvelde H, Peersman W, Lenoir M, Smits-Engelsman B, Henderson SE (2007). The movement assessment battery for children: similarities and differences between 4- and 5-year-old children from Flanders and the USA. Ped Phys Ther.

[16] Niemeijer AS, van Waelvelde H, Smits-Engelsman BC (2015). Crossing the North Sea seems to make DCD disappear: cross-validation of movement assessment battery for Children-2 norms. Hum Mov Sci.

[17] Tripathi R, Joshua AM, Kotian MS, Tedla JS (2008). Normal motor development of Indian children on Peabody developmental motor Scales-2 (PDMS-2). Ped PhysTher.

[18] World Health Organization. International classification of functioning, disability and health—children & youth version. Geneva; 2007.

[19] Livesey D, Lum Mow M, Toshack T, Zheng Y (2010). The relationship between motor performance and peer relations in 9- to 12-year-old children. Child Care Health Dev.

[20] Caspersen CJ, Powell KE, Christenson GM (1985). Physical activity, exercise, and physical fitness: definitions and distinctions for health-related research. Public Health Rep.

[21] Corbin CB, Pangrazi RP, Franks BD (2000). Definitions: Health, fitness, and physical activity. President's Council on Physical Fitness and Sports Research Digest.

[22] Smits-Engelsman BCM, Bonney E, Neto JLC, Jelsma LD (2020). Feasibility and content validity of the PERF-FIT test battery to assess movement skills, agility and power among children in low-resource settings. BMC Public Health.

[23] Smits-Engelsman B, Cavalcante Neto JL, Draghi TTG, Rohr LA, Jelsma LD (2020). Construct validity of the PERF-FIT, a test of motor skill-related fitness for children in low resource areas. Res Dev Disabil.

[24] Bruton A, Conway JH, Holgate ST (2000). Reliability: what is it and how is it measured?. Phys..

[25] Warburton DE, Gledhill N, Jamnik VK, Bredin SS, McKenzie DC, Stone J, Charlesworth S, Shephard RJ (2011). Evidence-based risk assessment and recommendations for physical activity clearance: consensus document 2011. Appl Physiol Nutr Metab.

[26] Smits-Engelsman BCM (2018). Performance and fitness battery for children.

[27] Mokkink LB, Terwee CB, Patrick DL, Alonso J, Stratford PW, Knol DL, Bouter LM, de Vet HC (2010). The COSMIN study reached international consensus on taxonomy, terminology, and definitions of measurement properties for health-related patient-reported outcomes. J Clin Epidemiol.

[28] Shrout PE, Fleiss JL (1979). Intraclass correlations: uses in assessing rater reliability. Psychol Bull.

[29] Cicchetti DV (1994). Guidelines, criteria, and rules of thumb for evaluating normed and standardized assessment instruments in psychology. Psychol Assess.

[30] Cicchetti D, Bronen R, Spencer S, Haut S, Berg A, Oliver P, Tyrer P (2006). Rating scales, scales of measurement, issues of reliability: resolving some critical issues for clinicians and researchers. J Nerv Ment Dis.

[31] Portney LG, Watkins MP (2009). Foundations of clinical research: applications to practice.

[32] Field A. Discovering statistics using IBM SPSS statistics, 2013; 5th edition. ISBN: 9781526419521 Sage Publications Ltd (UK).

[33] Fern EF, Monroe KB (1996). Effect-size estimates: issues and problems in interpretation. J Consum Res.

[34] Bland JM, Altman DG. Statistical methods for assessing agreement between two methods of clinical measurement. Lancet. 1986:307–10. 2868172

[35] Weir JP (2005). Quantifying test-retest reliability using the intraclass correlation coefficient and the SEM. J Strength Cond Res.

[36] De Vet HC, Terwee CB, Mokkink LB, Knol DL. Measurement in medicine: a practical guide: Cambridge University Press; 2011.

[37] Atkinson G, Nevill AM (1998). Statistical methods for assessing measurement error (reliability) in variables relevant to sports medicine. Sports Med.

[38] Kottner J, Audige L, Brorson S, Donner A, Gajewski BJ, Hróbjartsson A, Roberts C, Shoukri M, Streiner DL (2011). Guidelines for reporting reliability and agreement studies (GRRAS) were proposed. Int J Nurs Stud.

[39] D’Olhaberriague L, Litvan I, Mitsias P, Mansbach HH (1996). A reappraisal of reliability and validity studies in stroke. Stroke..

[40] De Vet HCW, Terwee CB, Knol DL, Bouter LM (2006). When to use agreement versus reliability measures. J Clin Epidemiol.

[41] Stratford PW, Goldsmith CH (1997). Use of the standard error as a reliability index of interest: an applied example using elbow flexor strength data. Phys Ther.

[42] Bartlett JW, Frost C (2008). Reliability, repeatability and reproducibility: analysis of measurement errors in continuous variables. Ultrasound Obstet Gynecol.

[43] Strainer DL, Norman GR, Cairney J (2014). Health Measurement Scales: A practical guide to their development and use 5 ed.

